# Effects of Different Saline-Alkaline Conditions on the Characteristics of Phytoplankton Communities in the Lakes of Songnen Plain, China

**DOI:** 10.1371/journal.pone.0164734

**Published:** 2016-10-17

**Authors:** Fengyang Sui, Shuying Zang, Yawen Fan, Huaxiang Ye

**Affiliations:** 1 Key Laboratory of Remote Sensing Monitoring of Geographic Environment, College of Heilongjiang Province, Harbin Normal University, Harbin, Heilongjiang, People’s Republic of China; 2 Key Laboratory of Botany, Harbin Normal University, Harbin, Heilongjiang, People’s Republic of China; Northeast Forestry University, CHINA

## Abstract

Many lakes located in the Songnen Plain of China exhibit a high saline-alkaline level. 25 lakes in the Songnen Plain were selected as research objects in this study. Water samples in these lakes were collected from June to August in 2008. Total Dissolved Solids (TDS) and Total Alkalinity (TA) were measured to assess the saline-alkaline level, and partial canonical correspondence analysis (CCA) was conducted as well. The results show that the majority of these lakes in the study area could be categorized into HCO_3_^−^-Na^+^-I type. According to the TDS assessment, of the total 25 lakes, there are 14 for freshwater, 7 for brackish water and 4 for saltwater; and the respective range of TA was from 0.98 to 40.52. The relationship between TA and TDS indicated significant linear relationship (R^2^ = 0.9292) in the HCO_3_^−^-Na^+^-I type lakes in the Songnen Plain. There was a general trend that cell density, genera richness and taxonomic diversity decreased with the increase of saline-alkaline gradient, whereas a contrary trend was observed for the proportion of dominant species. When the TDS values were above 3×10^3^mg/L and the TA values were above 15mg/L, there was a significant reduction in cell density, genera richness and biodiversity, and their corresponding values were respectively below 10×10^6^ (ind/L), 15 and approximately 2.5. Through the partial canonical correspondence analysis (CCA), 10.7% of the genera variation was explained by pure saline-alkaline variables. *Cyclotella meneghiniana*, *Melosira ambigua* and *Melosira granulate* were found to become the dominant species in most of these lakes, which indicated that there may be rather wide saline-alkaline niches for common dominant species. About one-quarters of the genera which have certain tolerance to salinity and alkalinity preferred to live in the regions with relatively higher saline-alkaline levels in this study.

## Introduction

At present, there are a number of problems of lakes in various parts of the globe, such as lower water level, lesser water area, and higher saline-alkaline level. Moreover, saline-alkaline environments of lakes and the resulting change in structure and function of aquatic ecosystem have become the focus of attention [1]. The group of lakes in the Songnen Plain is the chief component of the lakes of Northeast Plain in China [2], and the lakes in the Songnen Plain provide a unique water environment for the study of phytoplankton communities. Located in the depression belt of Songliao area, the Songnen Plain began to subside chronically since the Mesozoic, its bedrock is embedded deeply [3, 4]. Its top is covered with Quaternary fluviolacustrine facies sediment with high salt saturation in the rock, and its water solution is alkali. The cations are mainly Na^+^_,_ and the anions are mainly HCO_3_^-^ among the salt components [4, 5]. With the essential characteristics of saline and alkaline, these lakes formed in a large-scale regional climate background and hydrological conditions, and surface geochemical actions are special [4].

Saline-alkaline level is a primary factor influencing the phytoplankton community, as different kinds of phytoplankton have their optimum saline- alkaline levels [1]. Phytoplankton biomass, cell diameter, and diatom density decreased seaward across the low salinity zone [6]. Along estuary gradients cyanobacteria and chlorophytes tend to appear in brackish waters [7, 8]. Dinoflagellates and diatoms favor mid-to-high salinities (10 ppt) [9]. Experimental studies have shown that *Prorocentrum* spp can grow over a broad salinity range [10]. The wide variation of salinity resulted in a highly dynamic submerged aquatic vegetation community [11]. The adaptability of algae to salinity and alkalinity is different because of the physiological difference in some species [12]. The species with a high salinity optimum usually have a quite high tolerance [13]. Salinity is negatively correlated with phytoplankton biomass [14, 15]. Redden & Rukminasari [16] reported phytoplankton in the lower basin of the Myall Lakes responded to increases in salinity, from 1.5–5.5 ppt, with a decrease in chlorophyll a and an increase in taxonomic diversity. The growth, lipid and triacylglyceride accumulation of *Dunaliella* were inhibited by high salinity [17]. The gross chemical and fatty acid composition of *Isochrysis* sp., *Nannochloropsis oculata*, and *Nitzschia* are significantly different at different saline levels [18]. The observed strong correlations of algal abundance and biomass with rainfall (positively) and alkalinity-salinity (negatively), probably suggest that hydrological control of the salinity is the major driving force for the seasonal variability of *Arthrospira fusiformis* [19].

Much of our current knowledge about the effects of salinity and alkalinity is based on the studies of hydrochemical characteristics of lakes in the Songnen Plain [2, 4, 20]. Surprisingly, few researches have been conducted on the effects of changes in salinity and alkalinity on phytoplankton communities for naturally occurring phytoplankton assemblages. XU Jinyu et al. [21] had taken Zhalong, Xianghai, Momoge and Chagan lake wetland protection zones as examples, investigating the species composition and amount change of phytoplankton. LUO Xinzheng et al. [22] reported 5 phyla and 35 genera of phytoplankton obtained from Yaopao Lake of Da’an Palaeochannel Area, which is one of the typical alkalescence lake wetlands in the Songnen Plain. SUI Fengyang’s study [23] showed the species composition, community structure and biodiversity analysis of phytoplankton of the 32 lakes in the Songnen Plain. In their studies, there is no focus on the analysis of effects of saline-alkaline levels on phytoplankton.

To date, most researches were experimentally conducted through increasing salinity or alkalinity to study the effects of levels of salinity or alkalinity on phytoplankton [16, 24–27]. This paper focuses on the use of environmental data measured from natural water of the lakes in the Songnen Plain, combined with the analysis of partial Canonical Correspondence Analysis to provide a relevant study of phytoplankton. The primary aims of the study were to reveal the impact of differences in the levels of salinity and alkalinity on phytoplankton assemblage structure and biodiversity, and to test the phytoplankton adaptability to salinity and alkalinity.

## Materials and Methods

### Study location and characteristics

The Songnen Plain, located in the middle and western part of the Northeast China, is an alluvial-diluvial plain by the Songhua River, the Nenjiang River and their tributaries [28]. The eastern, northern and western parts of the plain are bordered on Changbai Mountains, Xiaoxing’anling Mountains and Daxing’anling Mountains respectively. The southern part is on the border of the watershed between Songhua River and Liaohe River. The Nenjiang River runs from the northwest to the middle part of the plain, and flows into the Songhuajiang River, and then flows into the northeast part in the Heilongjiang Province [29]. Also, there are many branch rivers originated from surrounding mountains. Inside the region, many closed flow areas and ephemeral rivers are distributed, which caused a lot of wetlands [30]. The climate, affected by continental monsoon, is classified as the transition of sub-humid and semi-arid [29]. Annual average temperature is about 4–5.5°C, and annual precipitation is about 400-500mm, 70%-80% of which is occupied by the precipitation from June to September [31].

**Fig 1:**
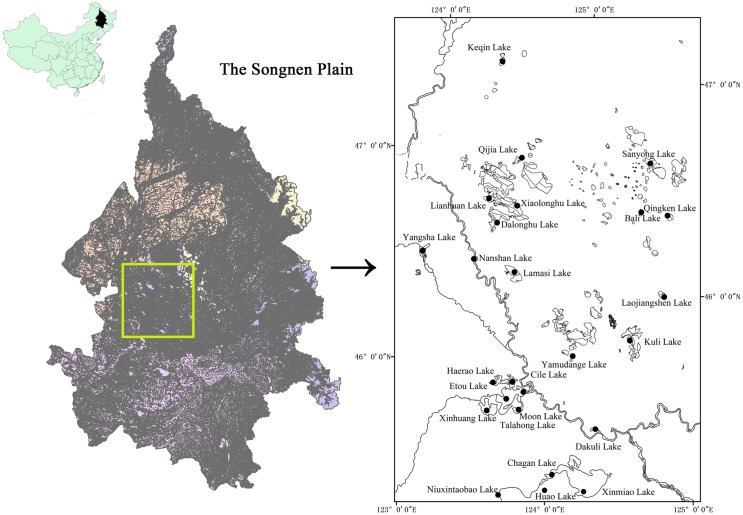
The study lakes are located in the Songnen Plain of Northeast China, and this figure is generated from USGS National Map Viewer (public domain): http://viewer.nationalmap.gov/viewer/.

The Songnen Plain is a low-lying plain, and the low-lying terrain makes the Songhua River, Nenjiang River and their tributaries form centripetal water systems in the Songnen Plain. According to the statistics, there are more than 700 lakes in this area, of which over 200 lakes basins cover an area of over 1 km^2^ each, and salt concentration is high in many lakes with alkali water [4, 32]. The lakes mainly distribute along the coasts of the Songhua River, the Nenjiang River and their tributaries. Based on the succession of distribution, environmental character, regional difference of the principal types and type combination, the lakes can be divided into six sub-regions. They are Daqing lake groups (r_1), Momoge lake groups (r_2), Sheli lake groups (r_3), Qian’an lake groups (r_4), Da’an lake groups (r_5) and Xiangwu lake groups (r_6) [33], and parts of these lakes from the first five sub-regions (r_1-r_5) are involved in this study. There are a total of 25 lakes were investigated in the present study (Fig 1), and the authority responsible for these lakes named Songliao Water Resources Commission of Ministry of Water Resources.

### Sample collection

Phytoplankton samples were collected from the 25 lakes in the Songnen Plain during June to August 2008. The quantity of sampling sites varied from lake to lake based on the area and the environmental conditions of each lake. A total of 77 samples were collected. The samples were collected from each site at a water depth of 0.5m using water samplers and then were preserved using Lugol’s solution (15ml Lugol’s solution was added into per 1000ml water sample). For quantitative analyses of phytoplankton communities, the mixed water was collected at each site and precipitated for 24-36h, the finally volume set to 30mL of concentrated sediments. A 0.1mL subsample of this finally concentrate was placed in a perspex counting chamber (Uwitec, Austrian) and enumerated under a light microscope at a magnification of 40× (Olympus BH×2, Japan). Mean figure of statistically values as biological density of each lake and 400 valves were counted from each sample. Because of Bacillariophyta can’t to be identified into species at a magnification of 40× (Olympus BH×2, Japan), so the total number of Bacillariophyta cell first to be counted, and then part of each sample was made to be permanent slides through acid treatment and centrifugation. The proportion of each taxa of Bacillariophyta sealed in permanent slides was calculated under a light microscope at a magnification of 100× oil lens (Olympus BH×2, Japan). Identification of taxa followed the volumes from Hu [34] and Krammer & Lange-Bertalot [35–38]. Meanwhile, the finally concentrate of each site was preserved in a sealed, shading and dry place.

According to the contribution of principal salt components to regional alkalization, the main hydrochemical variables can be classified into two groups, i.e., fundamental variables and derivative variables. Fundamental variables, such as pH and eight kinds of major ions (K^+^, Na^+^, Ca^2+^, Mg^2+^, Cl^-^, SO_4_^2-^, CO_3_^2-^ and HCO_3_^-^) [4]. Water temperature and pH at each site were measured in filed using portable multi-parameter analyzer (DZB-718-A, China), and the average water temperature and pH of each lake were calculated, eight kinds of major ions were obtained from the references (Yao et al.) [2]. Derivative variables, such as Total Dissolved Solids ($\text{TDS}=[{\text{K}}^{+}]+[\text{N}{\text{a}}^{+}]+[\text{C}{\text{a}}^{2+}]+[\text{M}{\text{g}}^{2+}]+[\text{C}{\text{l}}^{-}]+[\text{S}{\text{O}}_{4}{}^{2-}]+[\text{C}{\text{O}}_{3}{}^{2-}]+\frac{1}{2}[\text{HC}{\text{O}}_{3}{}^{-}]$) [39] and Total Alkalinity (TA = [CO_3_^2−^] + [HCO_3_^−^]) [40]. The two derivative variables indicate combination situation of hydrochemical factors in salt marsh water body [4, 41] and they can also be used to assess the levels of salinity and alkalinity.

### Data analysis

Biodiversity, which was expressed by Shannon-Wiener’s index, was calculated with the following equations [42,43].

$$H\prime =-\sum p_{i}(\text{ln }p_{i})$$

Where,

*H’* is Shannon-Wiener index, *p*_*i*_ = *n*_*i*_/*N*, *n*_*i*_ = the individual number of genera i; *N* = the total number of genera.

To investigate the relationship between phytoplankton and environmental characters especially the saline-alkaline conditions, a partial canonical correspondence analysis (CCA) was performed [44]. It was necessary to create tables of phytoplankton genera and environmental data, and the data should be standardized, including the genera data should appear in at least two lakes and the environmental data were performed on lg(X+1) transformed. In this paper, the calculation process and drawing were conducted using Canoco for Windows 4.5. The statistical analyses among TDS, TA and H’ values were carried out via Excel 2010.

## Results

### Environment characteristics

The environment characteristics of lakes in the Songnen Plain are showed in Table 1. The saline-alkaline level of these lakes was measured through TDS and TA. Among the 25 lakes in this study, there were 14 lakes’ TDS values are < 1×10^3^ mg·L^-1^, 7 lakes’ TDS values are > 1×10^3^ mg·L^-1^ and < 3×10^3^ mg·L^-1^, 4 lakes’ TDS values are > 3×10^3^ mg·L^-1^ and < 10×10^3^ mg·L^-1^, these lakes are classified into freshwater, brackish water and saltwater respectively. The highest TA (namely 40.52) was observed in Etou Lake, while the lowest was in Bali Lake. The pH values showed weak alkaline (> 8.0) in these study lakes except Cile Lake (namely 7.80) and Bali Lake (7.07). The range of temperature in 25 lakes is from 20.86°C to 29.35°C in summer.

**Table 1:** Environment characteristics of the lakes in the Songnen Plain in 2008.

Lakes No.	Lake name	pH	WT(°C)	TA(mmol/L)	TDS(mg/L)	TDS assessment	Hydrochemical type	Regionalization
1	Etou Lake	8.99	25.98	40.52	3158.88	Saltwater	HCO_3_^−^-Na^+^-I	r_2
2	Haerao Lake	8.37	20.86	2.89	181.06	Freshwater	HCO_3_^−^-Na^+^-I	r_2
3	Cile Lake	7.80	21.45	3.55	232.20	Freshwater	HCO_3_^−^-Na^+^-I	r_2
4	Yangsha Lake	9.07	22.57	21.13	1628.44	Brackish water	HCO_3_^−^-Na^+^-I	r_2
5	Niuxintaobao Lake	9.47	22.54	6.79	652.09	Freshwater	HCO_3_^−^-Na^+^-I	r_3
6	Xinhuang Lake	9.39	23.45	5.72	489.65	Freshwater	HCO_3_^−^-Na^+^-I	r_2
7	Moon Lake	8.46	24.46	3.39	234.75	Freshwater	HCO_3_^−^-Na^+^-I	r_2
8	Talahong Lake	9.02	26.11	15.28	1315.76	Brackish water	HCO_3_^−^-Na^+^-I	r_2
9	Dakuli Lake	9.06	23.08	2.59	397.49	Freshwater	HCO_3_^−^-Na^+^-I	r_5
10	Chagan Lake	9.09	24.00	10.27	835.36	Freshwater	HCO_3_^−^-Na^+^-I	r_5
11	Huao Lake	9.29	23.95	34.54	8209.82	Saltwater	Cl^−^-Na^+^-I	r_4
12	Xinmiao Lake	8.36	25.25	3.87	259.21	Freshwater	HCO_3_^−^-Na^+^-I	r_5
13	Keqin Lake	8.64	24.39	6.49	338.49	Freshwater	HCO_3_^−^-Na^+^-I	r_1
14	Nanshan Lake	9.02	25.73	8.41	485.65	Freshwater	HCO_3_^−^-Na^+^-I	r_1
15	Lianhuan Lake	8.87	25.13	11.57	739.75	Freshwater	HCO_3_^−^-Na^+^-I	r_1
16	Lamasi Lake	8.76	23.53	4.58	253.45	Freshwater	HCO_3_^−^-Na^+^-I	r_1
17	Dalonghu Lake	8.88	25.22	3.87	248.61	Freshwater	HCO_3_^−^-Na^+^-I	r_1
18	Qijia Lake	8.94	26.55	8.85	650.28	Freshwater	HCO_3_^−^-Na^+^-I	r_1
19	Xiaolonghu Lake	9.81	26.05	26.74	2313.13	Brackish water	HCO_3_^−^-Na^+^-I	r_1
20	Qingken Lake	9.20	26.99	30.49	3025.60	Saltwater	HCO_3_^−^-Na^+^-I	r_1
21	Bali Lake	7.07	27.76	0.98	3997.75	Saltwater	SO_4_^2−^-Na^+^-II	r_1
22	Sanyong Lake	9.06	29.35	16.34	1301.85	Brackish water	HCO_3_^−^-Na^+^-I	r_1
23	Laojiangshen Lake	9.38	28.46	16.73	2099.27	Brackish water	HCO_3_^−^-Na^+^-I	r_1
24	Yamudange Lake	9.30	25.35	25.34	1676.66	Brackish water	HCO_3_^−^-Na^+^-I	r_1
25	Kuli Lake	9.42	24.59	10.40	1102.07	Brackish water	HCO_3_^−^-Na^+^-I	r_1

A large percentage of the characteristic of water saline-alkaline was contributed by the major cations and anions, and the hydrochemical type of water was determined by the proportion and relationship of major ions (K^+^, Na^+^, Ca^2+^, Mg^2+^, Cl^-^, SO_4_^2-^, CO_3_^2-^ and HCO_3_^-^). According to O. A. Arliekin's classification [20], it is found that the majority of these lakes in study area were categorized into HCO_3_^−^-Na^+^-I type, while Huao Lake was categorized into Cl^−^-Na^+^-Itype and Bali Lake was categorized into SO_4_^2−^−Na^+^-II type.

**Fig 2:**
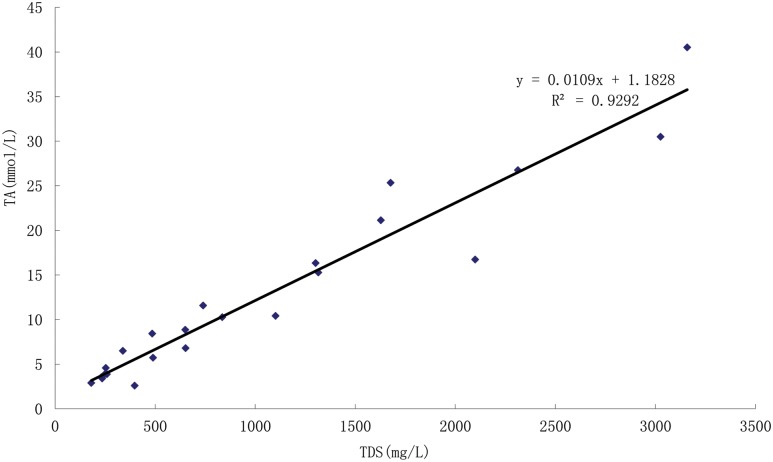
Relationship between TA (mmol/L) and TDS (mg/L) of water from the lakes in the Songnen Plain (two pairs of extreme values of Huao Lake and Bali Lake had been removed).

The relationship between TA and TDS of water from 25 lakes in the Songnen Plain is analyzed, which demonstrated significant linear (R^2^ = 0.9292) when two pairs of extreme values of Huao Lake and Bali Lake had been removed (Fig 2). Considering the hydrochemical types of Huao Lake (Cl^−^-Na^+^-I) and Bali Lake (SO_4_^2−^−Na^+^-II) are both different from the other 23 lakes’ (HCO_3_^−^-Na^+^-I), which can be inferred that the higher value of TDS might be related to the higher concentration of chloride ion in Huao Lake, and the reason of the higher level of TA value might be due to the more content of sulfate ion in Bali Lake. Consequently, it is deduced that the values of TA increased with the increasing values of TDS in the lakes of HCO_3_^−^-Na^+^-Itype in the Songnen Plain.

### Phytoplankton communities

There are abundant phytoplankton in the lakes of Songnen Plain in summer of 2008, and the sum of 232 taxa were discovered, which includes 6 phyla, 8 classes, 16 orders, 27 families, 51 genera, 199 species, 29 varieties, and 4 forms. The cell density and Genera richness of phytoplankton collected from every lake are counted (Table 2), and it shows that the phytoplankton communities of the lakes in the Songnen Plain are provided with less taxa and more abundance. Comparing with other phytoplankton, Bacillariophyta is the major composition of the species and also the major amount of phytoplankton, followed by Chlorophyta. The community composition of phytoplankton might be classified as Bacillariophyta-Chlorophyta type in the Songnen Plain in summer.

**Table 2:** Characteristics of phytoplankton communities of the lakes in the Songnen Plain in 2008.

Lakes No.	Lake name	Cell density (ind/L)	Genera richness	Dominant species	Proportion of dominant species	H’ value
1	Etou Lake	2.17×10^6^	4	*Cyclotella meneghiniana*	53.85%	1.16
2	Haerao Lake	8.86×10^6^	14	*Melosira ambigua*	11.29%	2.49
3	Cile Lake	6.14×10^6^	10	*Mougeotia* sp.	13.95%	2.21
4	Yangsha Lake	2.83×10^6^	5	*Microcystis flos-aquat*	29.41%	1.56
5	Niuxintaobao Lake	8.11×10^6^	15	*Melosira ambigua*	12.33%	2.49
6	Xinhuang Lake	1.27×10^7^	28	*Anabaenopsis circularis*	21.35%	2.92
7	Moon Lake	1.60×10^7^	26	*Nostoc kihlmeni*	10.16%	2.85
8	Talahong Lake	1.67×10^6^	4	*Anabaenopsis circularis*	30.00%	1.28
9	Dakuli Lake	5.71×10^6^	7	*Cymbella cistula*	12.20%	1.83
10	Chagan Lake	1.90×10^7^	25	*Anabaena cylindrica*	8.77%	2.97
11	Huao Lake	6.71×10^6^	10	*Tetrastrum staurogeniaeforme*	40.43%	1.81
12	Xinmiao Lake	1.23×10^7^	26	*Melosira ambigua*	9.30%	2.91
13	Keqin Lake	6.29×10^6^	8	*Melosira ambigua*	36.36%	1.83
14	Nanshan Lake	1.07×10^7^	26	*Ceratium hirundinella*	17.33%	3.00
15	Lianhuan Lake	7.54×10^6^	22	*Melosira granulata*	19.32%	1.95
16	Lamasi Lake	1.57×10^7^	21	*Melosira granulata*	24.55%	2.46
17	Dalonghu Lake	1.27×10^7^	20	*Melosira ambigua*	8.99%	2.63
18	Qijia Lake	9.57×10^6^	17	*Melosira granulata*	25.37%	2.52
19	Xiaolonghu Lake	6.71×10^6^	8	*Cyclotella meneghiniana*	19.15%	2.03
20	Qingken Lake	7.71×10^6^	11	*Melosira granulata*	25.93%	2.17
21	Bali Lake	5.67×10^6^	4	*Euglena pisciformis*	47.06%	1.19
22	Sanyong Lake	6.14×10^6^	6	*Cyclotella meneghiniana*	34.88%	1.51
23	Laojiangshen Lake	6.29×10^6^	14	*Fragilaria unla* var. *acus*	13.64%	2.53
24	Yamudange Lake	8.57×10^6^	10	*Melosira granulata*	28.33%	2.08
25	Kuli Lake	1.33×10^7^	19	*Cyclotella meneghiniana*	12.90%	2.69

All the dominant species in different lakes were counted (Table 2), *Cyclotella meneghiniana*, *Melosira ambigua* and *Melosira granulate* were found in many lakes and accounted for a large proportion in their respective communities of the lakes.

### Effects of saline-alkaline conditions on phytoplankton

**Fig 3:**
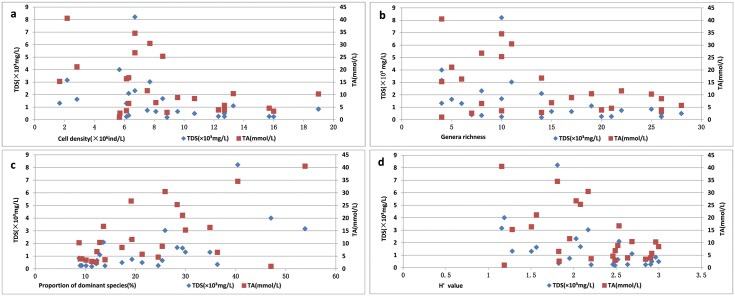
Variations of cell density (a), genera richness (b), proportion of dominant species (c) and H’ value (d) at different levels of TDS (mg/L) and TA (mmol/L) of water from the lakes in the Songnen Plain.

There is a general trend that cell density (Fig 3a), genera richness (Fig 3b) and H’ value (Fig 3d) decreased with the increase of saline-alkaline gradient, while the proportion of dominant species (Fig 3c) increased with the rising saline-alkaline gradient. However, no clear correlation shows between the saline-alkaline level and the characteristics of phytoplankton communities, due to the distribution of species and quantities of the phytoplankton is not simply determined by the saline-alkaline levels, other conditions such as geography, climate and water depth also have important influence to the survival and distribution of phytoplankton.

Generally, negative effects of saline-alkaline enhancement are observed for cell density, genera richness and phytoplankton diversity which was supported by the Shannon-Wiener index (H’) results in the communities. The distribution trend of cell density (Fig 3a), genera richness (Fig 3b) and phytoplankton diversity (Fig 3d) of the lakes are roughly opposite to the proportion of dominant species (Fig 3c) in this study. Additionally, the proportion of dominant species can response the evenness of the community to some extent. The higher the proportion of dominant species is, the lower the evenness of the community is. Based on the above, it can also be seen that the higher cell density, genera richness, and the evenness between species are, the higher phytoplankton diversity is in this study.

Fig 3 shows most of the TDS values of the lakes occurred below 3×10^3^ mg/L, and most of the TA values of the lakes were below 15 mg/L in the Songnen Plain. When the TDS values are less than 3×10^3^ mg/L and the TA values re less than 15 mg/L, the distribution ranges of their corresponding cell density, genera richness, proportion of dominant species and diversity value (H’) are relatively wide. Comparatively, when the TDS values are above 3×10^3^ mg/L and the TA values are above 15 mg/L, the values of their corresponding cell density, genera richness and diversity (H’) are respectively below 10×10^6^ (ind/L), 15 and approximately 2.5, and the values of the corresponding proportion of dominant species are above approximately 13. It can be demonstrated that the values of cell density, genera richness and diversity (H’) of phytoplankton are relatively lower, and the values of proportion of dominant species are relatively higher when the contents of TDS and TA in the lakes were at higher level.

### Partitioning the variation of genera matrix

The analyses were made using CCA after DCA analysis had been performed (the length of the longest axis of DCA analysis was > 3), indicating that phytoplankton of the lakes in the Songnen Plain showed unimodal responses to the environmental gradients of their biotopes in summer. The four analyses gave the following global results:

1. CCA of the genera matrix constrained by the saline-alkaline matrix: sum of all canonical eigenvalues = 0.343.
2. CCA of the genera matrix, constrained by the matrix of the other environmental variables (pH, WT and Regionalization): sum of all canonical eigenvalues = 0.893.
3. like (1), after removing the effect of the matrix of the other environmental variables (pH, WT and Regionalization): sum of all canonical eigenvalues = 0.256.
4. like (2), after removing the effect of the saline-alkaline matrix: sum of all canonical eigenvalues = 0.807

The sum of all eigenvalues in a correspondence analysis of the genera matrix is 2.389. Thus the percentage of the total variation of the genera matrix accounted for by each step of the analysis is obtained as follows:

step (1): (0.343/2.389)×100% = 14.4%
step (2): (0.893/2.389)×100% = 37.4%
step (3): (0.256/2.389)×100% = 10.7%
step (4): (0.807/2.389)×100% = 33.8%

The overall amount of explained variation (in percentage of the total variation of the genera matrix) is obtained either by summing the results of steps (1) and (4), or those of (2) and (3): 14.4%+33.8%≈37.4%+10.7% = 48.2%.

The whole variation of the genera matrix can be partitioned as follows: genera variation explained by pure saline-alkaline variables [step (3)]: 10.7%; genera variation explained by the intersection of the saline-alkaline and the other environmental variables (pH, WT and Regionalization) [step (1)-step (3), or step (2) -step (4)]: 3.6%; genera variation explained by the other environmental variables(pH, WT and Regionalization) that was not shared by the saline-alkaline variables [step (4)]: 33.8%; unexplained variation and stochastic fluctuations: 100%−48.2% = 51.8%.

### Effects of pure saline-alkaline conditions on phytoplankton

**Fig 4:**
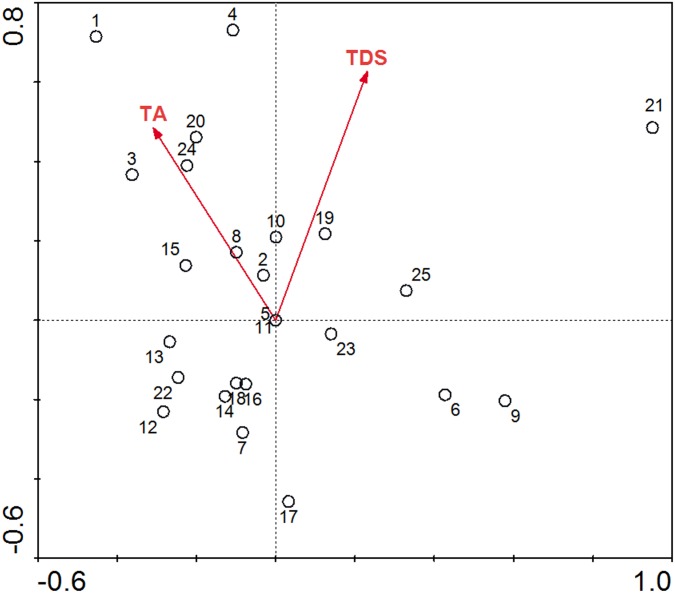
Partial canonical correspondence analysis (CCA) between saline-alkaline parameters (TA and TDS) and the 25 lakes’ samples in the Songnen Plain. No.1 to No.25 represents the samples of the 25 lakes respectively, and the corresponding relations of number and the lakes’ name are shown in Table 1.

**Fig 5:**
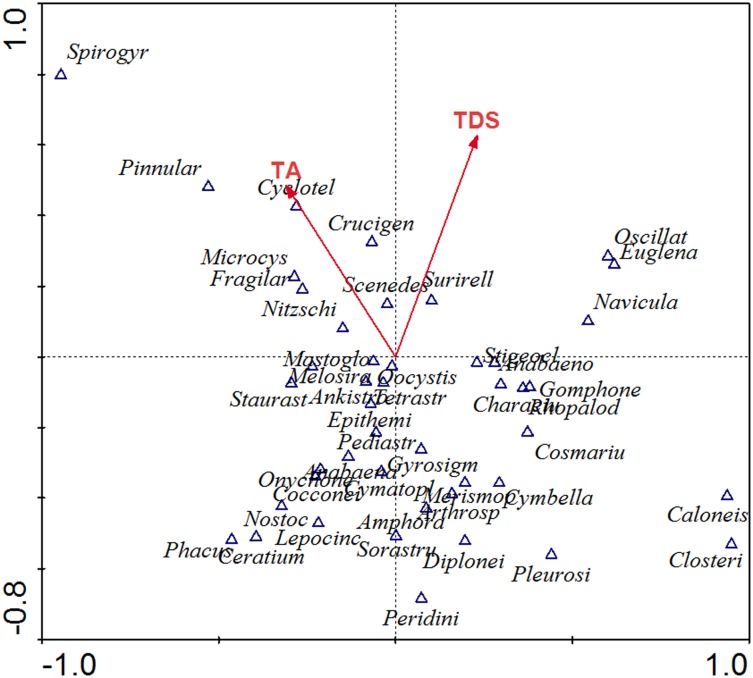
Partial canonical correspondence analysis (CCA) between phytoplankton genera and saline-alkaline parameters (TA and TDS) in the Songnen Plain.

The genera variation explained by pure saline-alkaline variables (TDS and TA) is performed by partial CCA, whose value is 10.7%, and the inflation factors are both < 10, which shows there is no strong collinearity among different data [45]. Phytoplankton assemblages and dominant taxa vary greatly in different lakes (Table 2). Partial CCA of all sites combined is undertaken to demonstrate how the saline-alkaline conditions influenced the phytoplankton community. The ecological characteristics of phytoplankton communities in different lakes are reflected through the positions of the 25 lakes’ samples in the border map (Fig 4), and the saline-alkaline niche of each genera is roughly reflected from Fig 5, which shows the genera scores of partial CCA.

In the lakes, Etou Lake (1), Xiaolonghu Lake (19), Sanyong Lake (22) and Kuli Lake (25) were dominated by *Cyclotella meneghiniana*, Haerao Lake (2), Niuxintaobao Lake (5), Xinmiao Lake (12), Keqin Lake (13) and Dalonghu Lake (17) were dominated by *Melosira ambigua*, Xinhuang Lake (6) and Talahong Lake (8) were dominated by *Anabaenopsis circularise*, Lianhuan Lake (15), Lamasi Lake (16), Qijia Lake (18), Qingken Lake (20) and Yamudange Lake (24) were dominated by *Melosira granulate*. It can be observed that the sample sites with the same dominant species are scattered in Fig 4, which indicates there are wider saline-alkaline niches for the dominant species.

Fig 5 indicates that the TA and the TDS are positively related to the second axis, and about three-quarters of the genera appeared in the range of negative semi-axes of the second axis, which reflects that most genera preferred to live in the regions of relatively lower saline-alkaline levels in this study. In comparison, the tolerance of the one-quarters of the genera for the TA and the TDS appears to be relatively higher, this part of the genera located in the range of positive semi-axes of the second axis were *Navicula*, *Nitzschia*, *Scenedesmus*, *Surirella*, *Fragilaria*, *Microcystis*, *Euglena*, *Oscillatoria*, *Crucigenia*, *Cyclotella*, *Pinnularia* and *Spirogyra*.

## Discussion

The hydrochemical characteristics are attributing to the factors of regional climate, hydrogeology, geomorphology and inappropriate human activities. The climate in the Songnen Plain is characterized dried in spring, heat and concentrated rainfall in summer, cool and larger temperature difference in autumn, and long and cold in winter [20]. Precipitation has the notable features, such as obvious stage and great variation. Hydrological dynamics of the study area is mainly controlled by precipitation and surface runoff, parts from the lakes are also affected by the groundwater, and furthermore, movement and enrichment of elements have consanguineous relationships with surface runoff [4]. TDS and TA of the lakes in the study area are higher than these in the humid areas, but far lower than these in the arid areas.

For the vast majority of lakes in this study, Na^+^ dominated the total cation and HCO_3_^-^ was the main component of anions, the water is alkaline and the hydrochemical type is soda-Na. Due to the difference between supply source and drainage modes and regional environment, the salt components and salt contents of lakes are quite different accordingly [4].

In the selected 25 lakes, Haerao Lake, Yangsha Lake, Moon Lake and Dalonghu Lake are now reservoirs, water recharge of Huao Lake is performed by taking the underground water from artesian wells around the lake, water recharge of Etou Lake, Lamasi Lake and Yamudange Lake just relay on precipitation and surface runoff. Cile Lake, Niuxintaobao Lake, Xinhuang Lake, Talahong Lake, Dakuli Lake, Chagan Lake, Xinmiao Lake, Keqin Lake, Nanshan Lake, Lianhuan Lake, Qijia Lake and Xiaolonghu Lake mainly relay on the Songhua River, Nenjiang River and its tributaries for water recharge. Sanyong Lake has become a running water lake since 1999 by the Nenjiang River-to-Anda water diversion project. Qingken Lake, Bali Lake, Laojiangshen Lake and Kuli Lake are located in Daqing detention basin, among them, Bali Lake is a pollutant lake, receiving sewage generated from a nearby factory. Additionally, the lakes which are surveyed in this study in the Songnen Plain are used for pisciculture nowadays except Bali Lake [31].

According to the records of lakes, Bali Lake has been contaminated due to the inflow of industrial wastewater from chemical plant and domestic sewage. The stench of Bali Lake could be smelt during the 2008 summer field survey, simultaneously, the pH (7.07) of Bali Lake was relatively low, which indicates that there may exist the emissions of sulfur-containing substance around the lake, resulting in low water pH, high sulphate content, low TA (total alkalinity) and low density of phytoplankton [2].

Among the 25 lakes in this study, there were 14, 7 and 4 lakes classified into freshwater, brackish water and saltwater, respectively. The saline-alkaline level in this study area exist the spatial inhomogeneity due to the environmental differences of small area. In general, the saline-alkaline level in noncontributing areas is higher than that in outflow ones; and the saline-alkaline level in the areas that has hydraulic connection with river is lower than that has no such relationship. Due to the differences of time and space of the water source, there is a stage characteristic of water saline-alkaline level with the change of water quantity and water level of the lakes, generally speaking, that is the water level is high, the water quantity is large and the saline-alkaline level is relatively low of the lakes in wet season, and the saline-alkaline level rises, the lakes shrink and even some lakes dry up in dry season.

In recent years, the water environment problems of salinization and alkalization of the Songnen Plain have become serious accompanied with population growth and the rapid development of industry, agriculture and economy [4, 46, 47], only in few large lakes that have relationship with rivers, such as Moon Lake, Lianhuan Lake and Chagan Lake, and the saline-alkaline level of this kind of lakes is relatively low [4]. The water quality data of the Songhua River from the late 1950s to the middle 1980s were analyzed by Chen Jingsheng [48], showed that the water quality alkalization occurred in Songhua River and in Nenjiang River (the content of Na^+^ and HCO_3_^-^ had increased). However, there are relatively fewer researches on hydrochemical analyse of the lakes in the Songnen Plain. Table 3 showed comparison of some lakes’ hydrochemical characteristics between the present study and some researches had been published. The saline-alkaline level of Huao Lake and Etou Lake were relatively higher due to there were no hydraulic connection with rivers. Additionally, compared with the previous studies, the level of TDS and the content of Na^+^ and HCO_3_^-^ in Huao Lake and Etou Lake had increased significantly. However, Chagan Lake, Moon Lake, Haerao Lake, Keqin Lake and Xinmiao Lake, the freshwater lakes, which had supply relationship with rivers, resulting in no obvious rise of the saline-alkaline level, even appeared the phenomenon of reduction of the saline-alkaline level in some lakes, such as Haerao Lake and Xinmiao Lake.

**Table 3:** Comparison of hydrochemical characteristics from various studies in 7 lakes in the Songnen Plain.

	Data Sources	Chagan Lake	Moon Lake	Huao Lake	Etou Lake	Haerao Lake	Keqin Lake	Xinmiao Lake
**Na**^**+**^ (mg/L)	Reference	297.30[4]	46.30[4]	2222.50[4]	388.00[20]	55.50[20]	35.45[20]	55.60[20]
	The present study	273.18	51.99	3142.70	1012.89	31.92	89.61	44.42
HCO_3_^-^ (mg/L)	Reference	501.02[4]	167.00[4]	1186.20[4]	2100.00[20]	228.00[20]	310.00[20]	270.00[20]
	The present study	559.70	203.87	1599.89	2289.72	176.46	391.70	236.26
TA (mmol/L)	Reference	10.06[4]	2.82[4]	24.41[4]	42.44[20]	3.97[20]	5.42[20]	4.49[20]
	The present study	10.27	3.39	34.54	40.52	2.89	6.49	3.87
TDS (mg/L)	Reference	887.00[4]	207.00[4]	7734.00[4]	2617.00[20]	242.45[20]	264.00[20]	275.00[20]
	The present study	835.36	234.745	8209.815	3158.88	181.06	338.49	259.21

The previous researches mentioned in this study were performed before 1999, and most of the data from references are the average values calculated during a certain period. Nevertheless, the samples of the present study are obtained in wet season, which may affect the saline-alkaline level of water. It is noteworthy that there is a phenomenon of concentrated precipitation and artificial water diversion for lakes’ storage in the Songnen Plain, thus there may exist quite differences of the results between different researches.

There are many factors affect phytoplankton growth and community structure in lakes, including salinity, alkalinity, light, nutrients, temperature and so on [7, 16, 49–51]. Salinity and alkalinity are the basic characteristics of hydrochemistry of the lakes in the Songnen Plain. Where salinities and alkalinities fluctuate, interspecific differences in tolerance of salinity and alkalinity of algae play a major role in structuring algae communities [52]. Some taxa, in particular *Cyclotella meneghiniana*, *Melosira ambigua and Melosira granulate*, tend to persist and even increased their proportion at higher saline-alkaline levels, owing to these dominant species have certain tolerance to salinity and alkalinity to certain extent. *Cyclotella meneghiniana* numerically dominate (12.90–53.85%) at different levels of TA (10.40–40.52mmol/L) and TDS (1102.07–3158.88mg/L) in several lakes; *Melosira ambigua* appeared as the dominant taxon (8.99–36.36%) in the lakes of different levels of TA (2.89–6.79mmol/L) and TDS (181.06–652.09mg/L); *Melosira granulate* dominate (19.32–28.33%) the assemblages from some lakes of different levels of TA (4.58–30.49mmol/L) and TDS (253.45–3025.60mg/L). As described in Krammer & Lange-Bertalot [37], *Cyclotella meneghiniana* is known to be halophilous and basophilous, and the taxa usually can be found in saltwater and brackish water; *Melosira ambigua* and *Melosira granulate* are the limnoplankton tend to favour low-salt environment. The results of this research about *Cyclotella meneghiniana*, *Melosira ambigua* and *Melosira granulate* roughly correspond with what Krammer & Lange-Bertalot [37] mentioned, only in addition that *Melosira granulate* has also been found as the dominant taxon in saltwater (Qingken Lake) and brackish water (Yamudange Lake), suggesting that there may be a wider salinity niche for *Melosira granulate*, although most occurrences occur in freshwater.

Cell density, genera richness, proportion of dominant species and taxonomic diversity (Shannon—Wiener index) showed no significant correlation with salinity (the TDS values) and alkalinity (the TA values) in this study. However, there was a significant reduction in cell density, genera richness and biodiversity when the TDS values were more than 3×10^3^mg/L and the TA values were larger than 15mg/L. Especially, the Shannon—Wiener index reflects the distribution, evenness and dominance of species at a particular site. A relatively lower diversity value indicates that some of the species which have competitive advantage in the specific environment present become dominant, resulting in the decrease of species evenness, then the diversity value is decreased, that’s the reason the distribution trend of diversity values of the lakes and the distribution trend of the proportion of the dominant species of the lakes are roughly opposite.

Some hydrochemical parameters related to nutrient concentrations show the alterations within the saline-alkaline gradient which are linked to the processes of mineralization and consumption by biota [43]. The increase of salinity and alkalinity will result in a reduction of growth rate and cell numbers of some freshwater or brackish water algae due to osmotic stress [52]. Different period of time was required for recovering from osmotic shock among different taxa [9]. In the lakes’ environment in the Songnen Plain, the species which have strong tolerance for salinity and alkalinity have competitive advantage. Taxa that contributed to the growth in number of genera observed at the higher saline-alkaline levels were *Navicula*, *Nitzschia*, *Scenedesmus*, *Surirella*, *Fragilaria*, *Microcystis*, *Euglena*, *Oscillatoria*, *Crucigenia*, *Cyclotella*, *Pinnularia* and *Spirogyra* (Fig 5).

A method for partitioning the variation of genera assemblages was used in this study, which allows one to measure the relative contribution of sets of explanatory variables by using eigenvalues of constrained and partial ordinations [44]. Operationally, it is based on the use of pre-existing methods of canonical ordination [53, 44] and computer programs.

Here, the saline-alkaline variables explain ≈ 15% of the variation in the genera matrix (step 1). Roughly one-third of this amount can also be predicted by the supplied function of the other environmental variables of the samples (step 1-step 3). This means that the genera and the saline-alkaline data have some fairly similar environmental factors, resulting from the interactions and common development of phytoplankton and environment during the process of lakes and biological evolution.

More than half of the total variation (51.8%) of the genera matrix remains unexplained, which is due to some factors (biotic or abiotic) weren't obtained in this study or to a large amount of stochastic variation remains unclear. It is worth noting, although there was no detailed investigation for light and nutrients in this study, the growth of phytoplankton and its distribution is also greatly influenced by light and nutrients which are usually crucial to phytoplankton and which may have co-effect on phytoplankton in the natural waters. Light is the prerequisite and guarantee for photosynthesis of phytoplankton, and light output have different effects on different species of phytoplankton, which mainly related to the factors such as photoinhibition of phytoplankton and utilization ratio of light energy. The impacts on the phytoplankton community by nutrients deserve studying. The change of the nutrient concentration, especially the ratio of nitrogen to phosphorus, in determining the composition of phytoplankton community plays an important role, which mainly affects the competition and the succession between phytoplankton to change the phytoplankton community. XIAO Haifeng reported that total nitrogen and total phosphorous in the lakes of Songnen Plain are generally high during June to August in 2008 [31], correspondingly, in our present study, *Cyclotella meneghiniana*, *Melosira ambigua* and *Melosira granulate* as the dominant species of this area are the common species usually living in eutrophication water.

## Conclusion

The hydrochemical characteristics in the Songnen Plain are affected by regional climate, hydrogeology, geomorphology and inappropriate human activities. Salinity and alkalinity which are measured by TDS and TA are the basic characteristics of hydrochemistry of the lakes in the Songnen Plain. Most lakes in this study could be categorized into HCO_3_^−^-Na^+^-Itype. Among the total 25 lakes, 14 of which are classified as freshwater, 7 as brackish water and 4 as saltwater separately according to the TDS assessment, the range of TA is from 0.98 to 40.52. The relationship between TA and TDS indicate significant linear relationship (R^2^ = 0.9292) in the HCO_3_^−^-Na^+^-I type lakes in the Songnen Plain. To compare with the previous researches, the level of TDS and the content of Na^+^ and HCO_3_^-^ in Huao Lake and Etou Lake had increased due to their no hydraulic connection with rivers, but there is also no obvious increase of the saline-alkaline levels of the lakes which had hydraulic connection with rivers.

There are 232 taxa of phytoplankton found in the 25 lakes with the characteristics of phytoplankton communities of less taxa and more abundance. Phytoplankton communities of the lakes in the Songnen Plain generally respond to increases in salinity and alkalinity, with decreases in cell density, genera richness and taxonomic diversity and an increase in proportion of dominant species. Increases in TDS values above 3×10^3^mg/L and TA values above 15mg/L results in significantly reduced values of cell density, genera richness and biodiversity.

The genera variation explained by pure saline-alkaline variables (TDS and TA) is performed by partial CCA, which value is 10.7%. The analyses of partial CCA demonstrated that *Cyclotella meneghiniana*, *Melosira ambigua* and *Melosira granulate* appeared in the lakes with different saline-alkaline levels as dominant species, owing to there may be rather wide saline-alkaline niches for the species. Furthermore, about one-quarter of the genera preferred to live in the environment of relatively higher saline-alkaline levels in this study, indicating these genera have strong tolerance to salinity and alkalinity.

## Supporting Information

S1 Appendix Average concentrations of cations and anions of water from 25 lakes in the Songnen Plain. (XLS)

S2 Appendix Distributions of phytoplankton genera in different lakes of the Songnen Plain. (XLS)

S3 Appendix Geographic coordinates of sampling sites from the Songnen Plain. (XLSX)

S1 Photo Innovation research Projects of Doctoral candidates of Harbin Normal University (HSDBSCX2015-13)-(1). (JPG)

S2 Photo Innovation research Projects of Doctoral candidates of Harbin Normal University (HSDBSCX2015-13)-(2). (JPG)

S3 Photo Innovation research Projects of Doctoral candidates of Harbin Normal University (HSDBSCX2015-13)-(3). (JPG)
