# Supplementary figures and images for: Effects of Different Saline-Alkaline Conditions on the Characteristics of Phytoplankton Communities in the Lakes of Songnen Plain, China
Source: PLoS One. 2016 Oct 17;11(10):e0164734. doi: 10.1371/journal.pone.0164734 (PMC5066945; doi:10.1371/journal.pone.0164734)

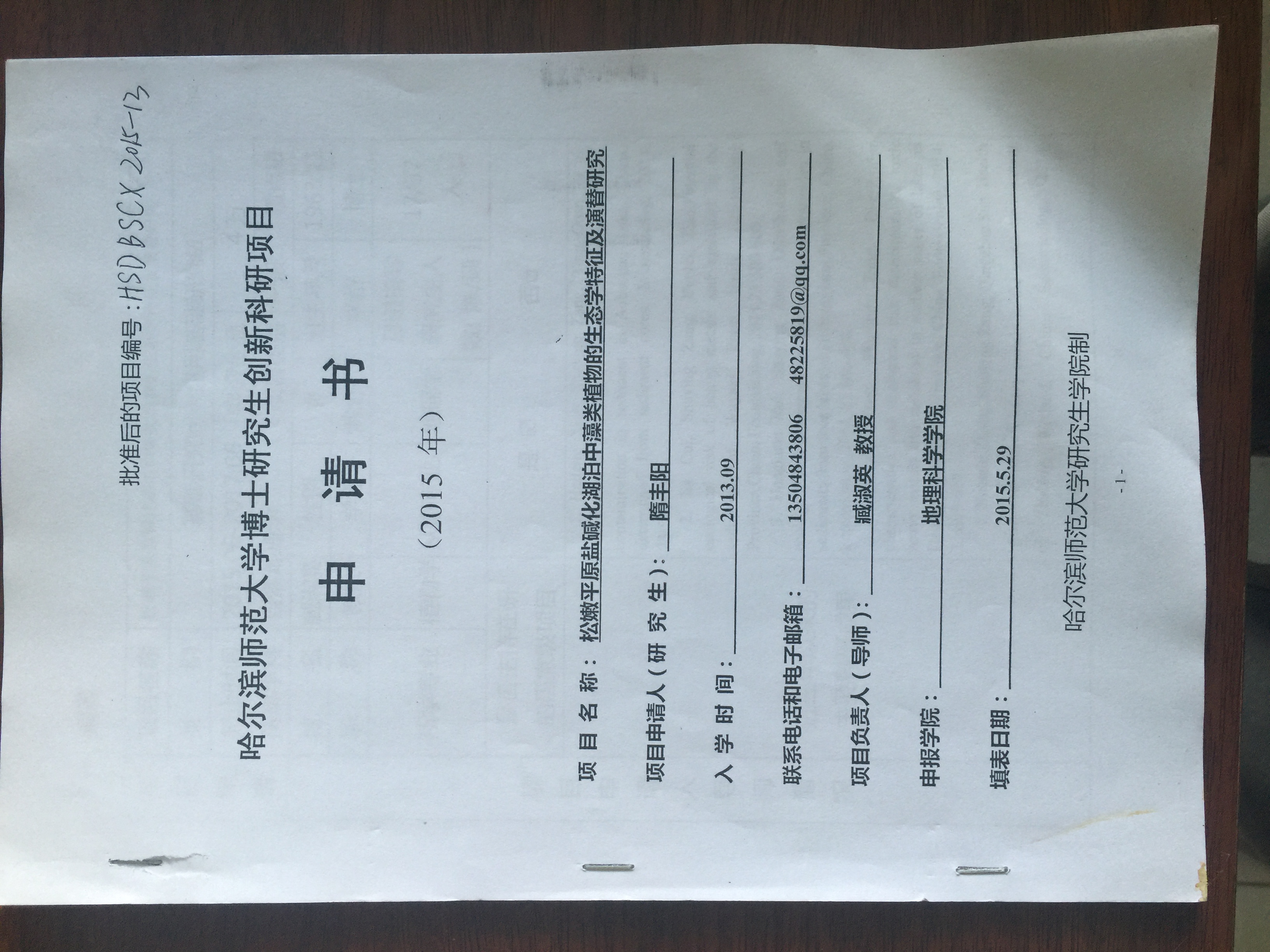

Supplement: S1 Photo — (JPG) [file pone.0164734.s004.JPG]

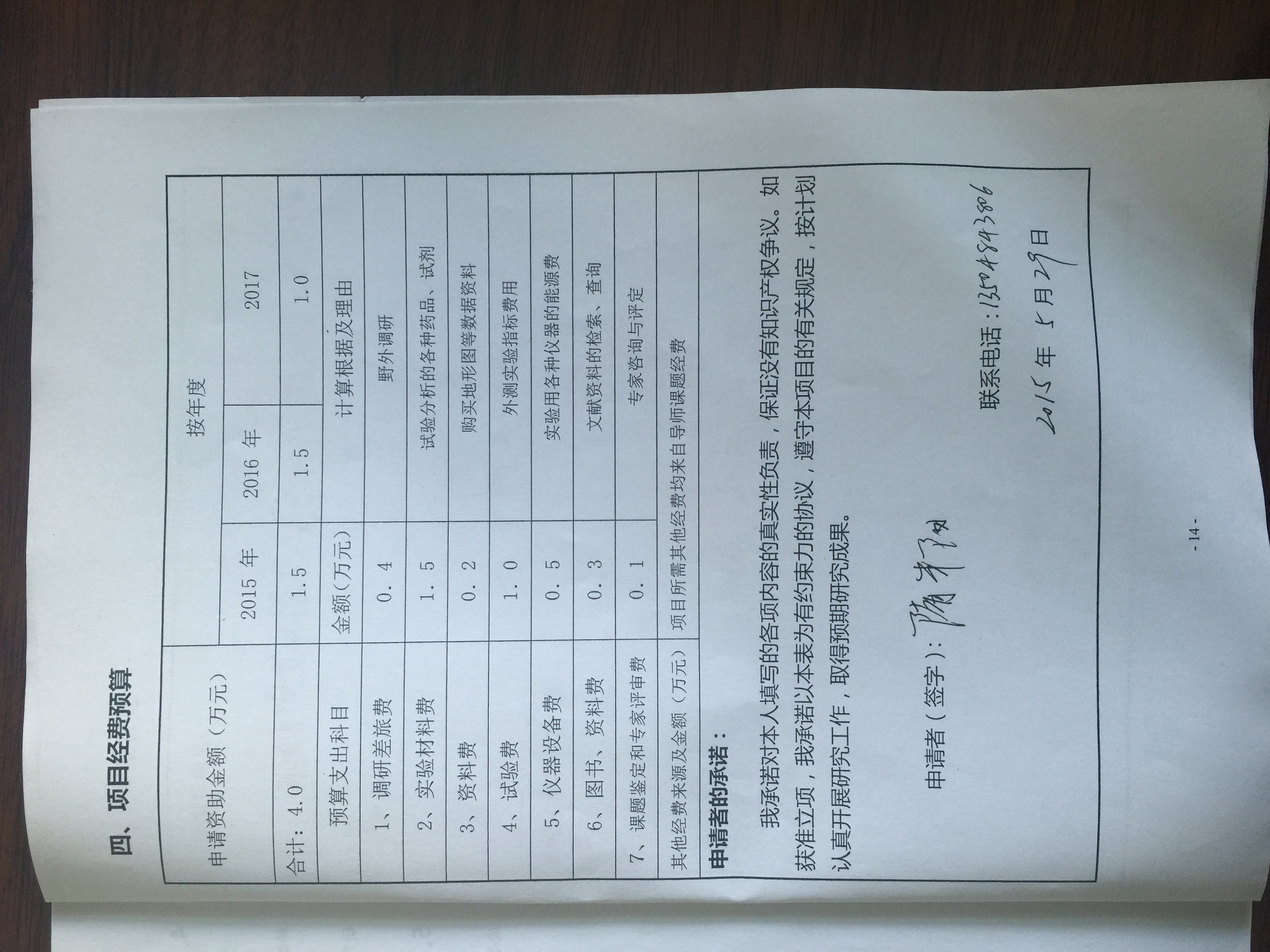

Supplement: S2 Photo — (JPG) [file pone.0164734.s005.JPG]

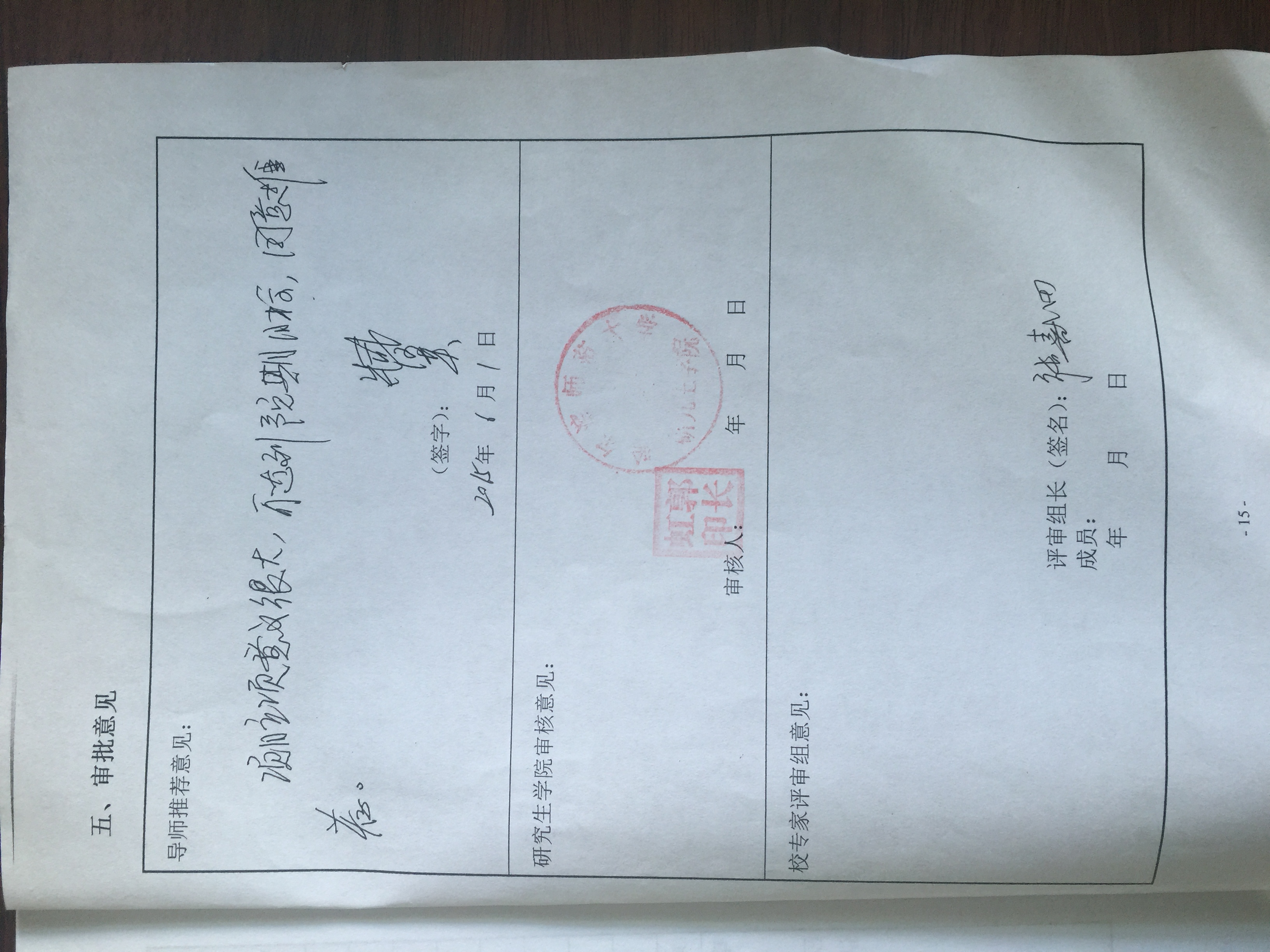

Supplement: S3 Photo — (JPG) [file pone.0164734.s006.JPG]
